# Philadelphia chromosome-positive acute myeloid leukemia successfully treated by allogeneic hematopoietic stem cell transplantation: A case report and review of the literature

**DOI:** 10.1097/MD.0000000000038110

**Published:** 2024-05-10

**Authors:** Zhichen Zhang, Xuan Wang, Jiaofeng Bai, Xiaolan Yang, Bianli Lian, Yuexia Zhang, Jin Kang, Yaozhu Pan

**Affiliations:** a Department of Hematology, The 940th Hospital of Joint Logistic Support Force of Chinese People’s Liberation Army, Lanzhou, China; b First School of Clinical Medicine, Ningxia Medical University, Yinchuan, China; c Department of Endocrinology, The 940th Hospital of Joint Logistic Support Force of Chinese People’s Liberation Army, Lanzhou, China.

**Keywords:** acute myeloid leukemia, allogeneic hematopoietic stem cell transplantation, BCR-ABL1, Philadelphia chromosome, tyrosine kinase inhibitor

## Abstract

**Rational:**

The Philadelphia chromosome (Ph) is seen in most patients with chronic myeloid leukemia and some patients with acute lymphoblastic leukemia. However, Ph-positive acute myeloid leukemia (Ph + AML) is a rare entity with a poor prognosis and a short median survival period. To date, there have been few clinical reports on this disease. And the treatment regimen of this disease has not been uniformly determined.

**Patient concerns:**

We report a case of a Ph + AML. A 32-year-old male who was admitted to our hospital with weakness for 2 months.

**Diagnosis:**

Philadelphia chromosome-positive acute myeloid leukemia.

**Interventions:**

The patient achieved complete remission by the administration of a tyrosine kinase inhibitor, combined with low-intensity chemotherapy and a B-cell lymphoma 2 inhibitor. Then, allogeneic hematopoietic stem cell transplantation (allo-HSCT) from his sister was successfully performed.

**Outcomes:**

The patient has been in a continuous remission state for 6 months after transplantation.

**Lessons:**

We reported a rare Ph + AML case, successfully treated with allo-HSCT. This case provided strong support for treating Ph + AML with allo-HSCT.

## 1. Introduction

The long-arm breakage and translocation of chromosomes 9 and 22 constitute the Philadelphia chromosome (Ph) and abnormal *BCR-ABL1* fusion gene. The *BCR-ABL1* fusion gene has been observed in 95% of patients with chronic myeloid leukemia (CML), 20% to 35% of adult patients with acute lymphoblastic leukemia (ALL), and 2% to 5% of pediatric patients with ALL.^[1]^ However, the incidence of Ph-positive acute myeloid leukemia (Ph + AML) is only 0.5% to 3%.^[2]^ The median survival of patients with Ph + AML was 9 months.^[3]^ Moreover, it has a low rate of complete remission (CR), a high risk of relapse, and an extremely poor prognosis.

There is no standard treatment regimen for this group of patients. In this report, a patient with Ph + AML was reported, complete hematologic remission was achieved with the administration of a tyrosine kinase inhibitor (TKI), combined with low-intensity chemotherapy and a B-cell lymphoma 2 inhibitor, following successfully by allogeneic hematopoietic stem cell transplantation (allo-HSCT). After transplantation, he maintained a continuous remission and minimal residual disease (MRD) negative state, we are glad to share the treatment experience of this rare case.

## 2. Case presentation

**Figure 1.**
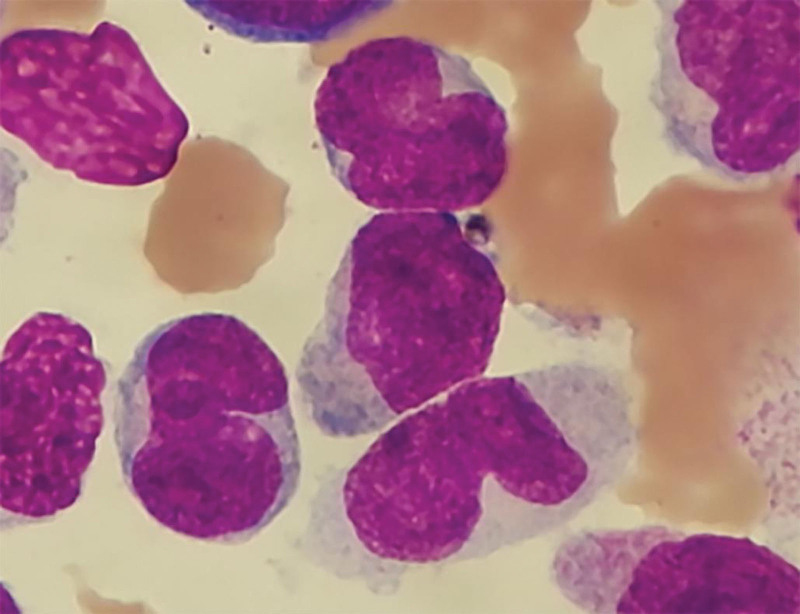
Bone marrow aspirate showing increased proportion of mononuclear cells (Wright stain, ×1000).

**Figure 2.**
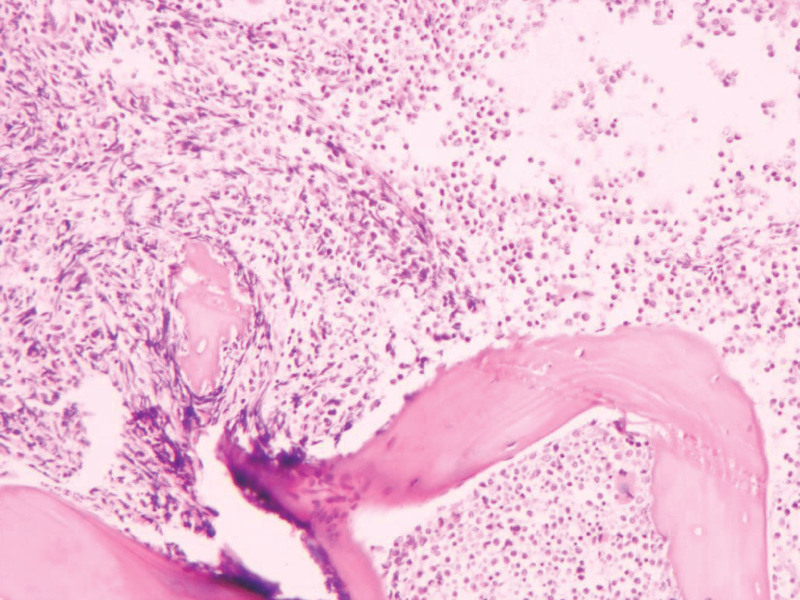
Bone marrow biopsy showing significantly active bone marrow tissue proliferation with diffuse proliferation of blast cells (HE, ×40).

On January 5, 2023, a 32-year-old male was admitted to our hospital for weakness, lasting 2 months. The patient had an unremarkable medical and family history. The physical examination showed signs of anemia. There was no noted tenderness in the sternum. The liver was not palpable, but the spleen was enlarged and palpable up to 4 cm in the subcostal region. Laboratory testing revealed: hemoglobin 63 g/L, white blood cells 78.43 × 10^9^/L, platelets 64 × 10^9^/L. The lactate dehydrogenase was elevated at 2001 U/L (normal 40–250 U/L). Abdominal ultrasound showed splenomegaly, 4 cm below the ribs. The bone marrow morphology showed that the primitive monocytes and premonocytes accounted for 62.4% (Fig. 1). Bone marrow biopsy revealed significantly active bone marrow tissue proliferation (about 90%) with diffuse proliferation of blast cells (Fig. 2). Flow cytometry results demonstrated an abnormally high myeloid blast cell population (69.1%) comprising of CD117, CD38, CD34, CD13, CD33, CD123, CD7, HLA-DR, CD56, and CD9. *BCR-ABL1* fusion gene (P210) and *WT1* gene were positive by RT-PCR, the WT1/ABL1% was 22.17%, while the IS BCR-ABL1/ABL1 (%) was 84.6%. Cytogenetic analysis revealed a karyotype of 46,XY,t(9;22)(q34;q11)[8]/49, idem, +2, +6, +8 [2]. The mutations of *FLT3, C-Kit, DNMT3A, NPM1*, and *CEBPA* were not detected. Based on the patient’s medical history and laboratory findings, he was diagnosed with Ph + AML-M5.

Induction chemotherapy was given on 9 January with the IA (idarubicin 20 mg, days 1–2; 10 mg, day 3; cytarabine 100 mg, days 1–7) regimen combined with flumatinib (600 mg qd). On March 1, the bone marrow smear showed 4.0% primitive monocytes and 8.0% premonocytes, the flow cytometry (FCM) showed that MRD was 8.69%, the IS BCR-ABL1/ABL1(%) was 58.8%. Partial remission was achieved, but the patient developed grade 4 myelosuppression for 47 days. Subsequently, his treatment was switched to a combination of venetoclax and azacitidine (venetoclax 100 mg, day 1; 200 mg, day 2; 400 mg, days 3–28; azacitidine 75 mg/m^2^, days1–7) and flumatinib (600 mg qd). One month later, he achieved CR and MRD was negative, 4 cycles of same therapy were repeated.

A whole evaluation was conducted, bone marrow morphology showed CR state, and MRD was negative by FCM. However, the IS BCR-ABL1/ABL1 (%) was 0.79%. Therefore, informed consent from the patient and his families, we decided to perform allo-HSCT for him. The donor was the patient’s 30-year-old sister, whose blood type and HLA type were fully matched (blood type A + for A+, HLA-matched 12/12). The conditioning regimen consisted of cytarabine 2 g/m^2^ (−9, −8 days), busulphan (Bu) 0.8 mg/kg q6h (−7, −6, −5 days), cyclophosphamide (Cy) 60 mg/kg (−4 days), antithymocyte globulin (ATG) 2.25 mg/kg (−3, −2 days), and MeCCNU 250 mg/m^2^ (−3 days). Donor peripheral blood stem cells were infused back with mononuclear cell count (MNC) of 9.23 × 10^8^/kg and CD34^+^ cell count of 2.01 × 10^6^/kg. Cyclosporine (CsA), mycophenolate mofetil, and short-course methotrexate (MTX) were administered as prophylaxis for graft-versus-host disease (GVHD). Neutrophil and platelet were engrafted at +13 days and +15 days, respectively.

By monthly monitoring of bone marrow morphology, MRD of FCM and quantification of *BCR-ABL1* fusion gene after transplantation, he maintained CR state, MRD negative, and negative *BCR-ABL1* fusion gene (Table 1); and short tandem repeat analysis showed complete chimerism with donor cells accounting for above 99.0% at +1, +3, +6 month. So far, followed up for 6 months, he was stable and healthy without GVHD, the flumatinib (400 mg qd) was also administered as maintenance treatment.

**Table 1:** The change of BCR-ABL1 fusion gene different treatment stages.

BCR-ABL1 fusion gene at diagnosis	BCR-ABL1 fusion gene after induction chemotherapy	BCR-ABL1 fusion gene before allo-HSCT	BCR-ABL1 fusion gene after allo-HSCT	BCR-ABL1 fusion gene at last follow-up
84.6%	58.8%	0.79%	Negative	Negative

allo-HSCT = allogeneic hematopoietic stem cell transplantation. BCR-ABL fusion gene was detected by real-time quantitative polymerase chain reaction. Results were considered negative if the IS BCR-ABL1/ABL1 ratio is ≤0.01%.

## 3. Discussion

Ph + AML is a rare entity that was initially believed to be derived from CML in myeloid blast crisis (CML-MBC). However, some studies have shown that primary Ph + AML presented with clinical and laboratory features, that were distinct from those of CML-MBC. The 2016 revision to the World Health Organization classification of myeloid neoplasms and acute leukemia added AML with *BCR-ABL1* as a new provisional category.^[4]^ According to previous literature,^[3,5–8]^ the 2 entities can be distinguished through the following characteristics. First, Ph + AML does not present with clinical or laboratory features of CML, such as marked splenomegaly and peripheral blood and/or bone marrow basophilia. Patients neither have a history of chronic or accelerated phase CML nor features of the chronic phase of CML in remission. Second, most Ph + AML cases are characterized by the co-expression of lymphoid markers. Third, the cytogenetic and molecular features are different between the 2 diseases. Additional cytogenetic abnormalities are more common in CML-MBC, while abnormalities in chromosome 7 occur more frequently in Ph + AML. *NPM1, FLT3, KIT*, and *IDH1/2* are commonly mutated in Ph + AML, while *ABL1, RUNX1, ASXL1*, and *IKZF1* mutations are common in CML-MBC. Fourth, return to a normal karyotype following induction chemotherapy is more common in Ph + AML patients, while the Ph persists in CML-MBC patients, even when in remission. In the present case, the patient had no history of CML or other hematological diseases. No basophilia and accompanied by expression of lymphoid antigens. The chromosomal karyotype returned to normal after TKI combination chemotherapy. These findings were strongly suggestive of primary Ph + AML. Although the patient’s splenomegaly at the time of his initial diagnosis suggested CML, Neuendorff et al^[5]^ reported that 21 of 126 Ph + AML patients presented with splenomegaly.

The administration of TKI, represented by imatinib, significantly improves the prognosis of CML patients. It also improves the remission rate in patients with Ph + AML. In a report by Soupir et al^[3]^ among 7 Ph + AML patients, treated with imatinib, 6 had a hematologic response. In particular, 1 patient achieved complete hematologic remission. However, the median duration of response was only 2.5 months. Kondo et al^[9]^ reported a patient with Ph + AML, that has been in complete molecular response (CMR) for more than 6 months. This patient received a daily dose of 400 mg of imatinib only. Flumatinib is a new second-generation TKI in China. A study by Yang et al^[10]^ indicated that flumatinib exerted a stronger mutation inhibition effect on ATP-binding regions (e.g., V299L, F317L, F317I), and its use for the treatment of patients with Ph + AML has not been reported in China. Early molecular remission was achieved in the patient within 3 months of treatment with flumatinib. Moreover, the fusion gene turned negative at 6 months. The medication was effective and well-tolerated and it can be used as an alternative drug for imatinib. However, long-term TKI administration can induce drug resistance among tumor cells, leading to disease relapse. On the other hand, Ph + AML is different from CML, which relies solely on *BCR-ABL1* fusion protein. Its pathogenesis is related to multiple factors, such as clonal transformation, complex genetic background, and multiple strikes. Therefore, the synergistic effect, caused by TKI therapy and the graft-versus-leukemia effect, allows patients to achieve long-term survival.

Allo-HSCT is an effective treatment option that improves the prognosis of patients with AML and achieves long-term relapse-free survival. Reboursiere et al^[7]^ reported 8 Ph + AML patients, who underwent TKI combined with allo-HSCT, with a median survival of 17 months. According to Min et al,^[11]^ the 5-year overall survival (OS) rate of 17 Ph + AML patients, treated with allo-HSCT, was 69.3%, while the median OS of 12 patients not treated with allo-HSCT was only 6.25 months. A study by Lazarevic et al^[12]^ included 57 *BCR-ABL1* positive AML patients, who underwent allo-HSCT. There were 26 MRD-positive patients before transplantation. After transplantation, 16 patients achieved MRD-negative status. Based on their results, patients under 50 years old with *BCR-ABL1* positive AML, treated with allo-HSCT in CR status, had a favorable prognosis. The patient’s real-time quantitative polymerase chain reaction showed *BCR-ABL1* fusion gene was positive before transplantation. This indicated a high risk of relapse. Therefore, allo-HSCT was conducted.

According to Lai et al^[13]^ despite the use of mycophenolate mofetil, CsA, and MTX triple regimens, the incidence of grade 2 to 4 acute GVHD (aGVHD) remained at 30% after matched sibling donor transplantation in patients at least 40 years old. In a study by Walker et al,^[14]^ 203 patients, who were treated via unrelated donor transplantation, were randomized into treatment groups of ATG plus standard GVHD prophylaxis (ATG group) and standard GVHD prophylaxis alone (no ATG group). There was no difference between the 2 groups in terms of the hematopoietic reconstitution time, non-relapse mortality, incidence of relapse, overall survival, and incidence of serious infections. However, the cumulative incidence of aGVHD in the ATG group was significantly lower than that of the no ATG group at 100 days (50% vs 65%, *P* = .012). Chang et al^[15]^ demonstrated that ATG effectively decreased the incidence of aGVHD after matched sibling donor transplantation without affecting the cumulative incidence of relapse or non-relapse mortality. The recommended ATG dose is 4.5 mg/kg. In this case, low-dose ATG (2.25 mg/kg × 2 d) was added to decrease the incidence of GVHD after transplantation and improve the patient’s quality of life. During the patient’s follow-up assessment 6 months after allo-HSCT, no signs of GVHD were observed.

Allo-HSCT is an effective treatment for AML. With the improvement of conditioning regimens and supportive treatments, the survival rate after transplantation continues to improve. However, the 2-year posttransplantation relapse rate of high-risk AML patients reached up to 37.5%.^[16]^ Moreover, the long-term survival rate significantly decreases in patients who experience a relapse. Therefore, providing maintenance therapy is crucial for posttransplantation patients with Ph + AML. TKI was reportedly the best maintenance drug for patients who underwent allo-HSCT. However, the specific TKI and its dose as well as the timing and duration of treatment remains controversial. In a study by Sun et al^[17]^ a 19-year-old female patient received imatinib at a dose of 300 mg/d 74 days after HSCT. This was maintained for 15 months after HSCT. The complete hematologic response, CMR, and complete cytogenetic response persisted 39 months after HSCT. For the optimal duration of posttransplantation imatinib maintenance therapy, Sun et al^[17]^ recommended at least 1 year. TKI can also be used for the treatment of posttransplantation relapse in Ph + AML patients. Lazarevic et al^[12]^ reported 9 patients, who experienced a relapse, treated with TKI. Five of the patients remained alive and in disease remission until the last follow-up. In this report, TKI therapy was restarted 2 months after transplantation. Provided that all indicators will be normal during the follow-up period, this treatment plan will be maintained for 1 year. We hypothesize that if the patient suffers a relapse, various therapeutic options, including the reduction of immunosuppression, donor lymphocyte infusion, hypomethylating agents, targeted agents, immunotherapy, and second transplantation, may be used in addition to the continued application of TKI.

## 4. Conclusion

In conclusion, Ph + AML is a heterogeneous acute leukemia with distinct biological characteristics compared to CML-MBC. The results of this study show that induction chemotherapy combined with TKI regimen is effective in the treatment of Ph + AML patients. Then bridging allo-HSCT can further improve CMR, and TKI maintenance therapy after transplantation may help to reduce relapse. However, this study also has its limitations. It is a case report with a short follow-up period. Thus, future studies with larger sample sizes and longer follow-up periods are necessary to validate the results.

## Acknowledgments

We thank the patient and his family for the publication of this study.

## Author contributions

**Conceptualization:** Zhichen Zhang, Xuan Wang, Jiaofeng Bai, Yaozhu Pan.

**Validation:** Zhichen Zhang.

**Writing – original draft:** Zhichen Zhang, Xuan Wang.

**Writing – review & editing:** Jiaofeng Bai, Yaozhu Pan.

**Data curation:** Xiaolan Yang, Bianli Lian.

**Formal analysis:** Yuexia Zhang, Jin Kang.

**Funding acquisition:** Yaozhu Pan.

**Supervision:** Yaozhu Pan.
